# Survival Outcomes of Ewing Sarcoma and Rhabdomyosarcoma by High- versus Low-Volume Cancer Centres in British Columbia, Canada

**DOI:** 10.3390/diagnostics13111973

**Published:** 2023-06-05

**Authors:** Sarah Yeo, Ursula Lee, Ying Hui Xu, Christine Simmons, Alannah Smrke, Ying Wang

**Affiliations:** 1 Faculty of Medicine, University of British Columbia, Vancouver, BC V6T 1Z3, Canada; 2 BC Cancer Surrey, Surrey, BC V3V 1Z2, Canada; 3 BC Cancer Vancouver, Vancouver, BC V5Z 4E6, Canada

**Keywords:** cancer, Ewing sarcoma, healthcare systems, oncology, outcomes research, overall survival, reference centres, rhabdomyosarcoma, sarcoma, treatment

## Abstract

Due to the rarity and complexity of treatment for Ewing sarcoma and rhabdomyosarcoma, studies demonstrate improved patient outcomes when managed by a multidisciplinary team at high-volume centres (HVCs). Our study explores the difference in outcomes of Ewing sarcoma and rhabdomyosarcoma patients based on the centre of initial consultation in British Columbia, Canada. This retrospective study assessed adults diagnosed with Ewing sarcoma and rhabdomyosarcoma between 1 January 2000 and 31 December 2020 undergoing curative intent therapy in one of five cancer centres across the province. Seventy-seven patients were included, 46 seen at HVCs and 31 at low-volume centres (LVCs). Patients at HVCs were younger (32.1 vs. 40.8 years, *p* = 0.020) and more likely to receive curative intent radiation (88% vs. 67%, *p* = 0.047). The time from diagnosis to first chemotherapy was 24 days shorter at HVCs (26 vs. 50 days, *p* = 0.120). There was no significant difference in overall survival by treatment centre (HR 0.850, 95% CI 0.448–1.614). Variations in care exist amongst patients treated at HVCs vs. LVCs, which may reflect differences in access to resources, clinical specialists, and varying practice patterns across centres. This study can be used to inform decisions regarding triaging and centralization of Ewing sarcoma and rhabdomyosarcoma patient treatment.

## 1. Introduction

Ewing sarcoma and rhabdomyosarcoma are two rare and clinically challenging diseases. With annual incident rates of only 0.7 cases per 1 million and 4.4 cases per 1 million, respectively, these highly aggressive malignancies require a comprehensive and collaborative approach to treatment [1,2]. Current management of Ewing sarcoma and rhabdomyosarcoma typically involves a combination of chemotherapy, radiation, and surgery [2]. 

Several internationally recognized clinical practice guidelines recommend that patients with sarcoma be managed at specialized, high-volume centres by a dedicated multidisciplinary team of expert pathologists, radiologists, surgeons, radiation oncologists, and medical oncologists, with review at multidisciplinary tumour boards [3,4,5]. This is especially important for patients with Ewing sarcoma and rhabdomyosarcoma, where due to the multimodal approach to therapy, close coordination between the various disciplines is paramount. The centralized management of patients with sarcoma has consistently been shown to have a significantly positive impact on patient outcomes, in terms of both local control and overall survival (OS) [6,7,8,9,10,11,12,13]. 

Based on this evidence, it is now internationally recognized that patients with rare cancers, such as Ewing sarcoma and rhabdomyosarcoma, should be treated at specialist high-volume centres [14]. Multiple countries, including the United Kingdom and Scandinavian countries, have already mandated that care of patients with sarcoma must occur at designated high-volume reference centres [15,16]. However, there are challenges in applying this standard to countries spanning a large geographical area or countries with a low population density. For instance, in British Columbia, Canada, the implementation of such policies may be less feasible than our European counterparts. The larger geographical area of the province of British Columbia presents unique challenges relating to the disparate distance patients must travel to access healthcare and higher travel-related financial burden, particularly for those living in rural and remote regions. While the majority of sarcoma surgeries in the province occur in Vancouver, which has the largest provincial academic tertiary care centres, radiation and systemic therapy for patients with sarcoma is given across five BC cancer centres throughout the province. It is a provincial standard of care that all patients with sarcoma are discussed at least once at the provincial multidisciplinary tumour board, which occurs weekly. Due to the distance that some patients need to travel to one of the five BC cancer centres that provide systemic and radiation therapy, the delivery of care for patients living in rural and remote regions is shared between general practitioner oncologists and oncology specialists in high-volume centres. For example, a patient with rhabdomyosarcoma can be given curative intent chemotherapy by a general practitioner oncologist at a city that is 1300 km away from the patient’s supervising medical oncologist, who is located at a high-volume centre in Vancouver. 

Given the mounting evidence for centralized care, it is important to evaluate the impact of the location of care for patients with Ewing sarcoma and rhabdomyosarcoma in British Columbia, Canada. Our study aims to explore the differences in outcomes of patients diagnosed with Ewing sarcoma and rhabdomyosarcoma in British Columbia based on the geographical location of diagnosis, treatment, and follow-up. 

## 2. Materials and Methods

In order to assess any impact of geographic location on progression-free survival or overall survival, a retrospective cohort study was conducted using data extracted from the British Columbia Cancer Sarcoma Outcomes Unit Database (BC SARCOU). Five cancer centres across British Columbia contribute data to the BC SARCOU. This database builds upon the BC Cancer Registry, a database of all new cancers diagnosed amongst British Columbians, and contains personal, demographic, diagnosis, and death information. This work was approved by the University of British Columbia BC Cancer Research Ethics Board, certificate H20-04050, prior to commencement. 

All adult patients aged 18 years or older at diagnosis, who were diagnosed with Ewing sarcoma or rhabdomyosarcoma between 1 January 2000 and 31 December 2020 and underwent curative intent therapy in British Columbia, were included in this study. These patients were identified using the BC Cancer Registry and BC SARCOU databases. Curative intent patients were selected to avoid heterogeneity of data in a small patient population. In addition, as healthcare in Canada is delivered on a provincial level with each province having its own regionalized healthcare system, our study focused on patients treated solely in British Columbia to avoid introducing biases from differing healthcare systems. Clinical tumour factors, treatment details, and follow-up data were manually extracted from the electronic patient record by retrospective chart review to supplement the demographic, diagnosis, and outcome data obtained from the BC SARCOU database. Baseline patient characteristics such as age, gender, age at diagnosis, Charlson Comorbidity Index (CCI) and Eastern Cooperative Oncology Group (ECOG) performance status, at diagnosis were recorded. Other variables collected included stage at diagnosis, presentation at a multidisciplinary sarcoma conference, treatment modality received, treatment regimen received, duration of treatment, date of recurrence if any, date of progression if any, and overall survival. Location was determined based on the initial site of oncology consultation. High-volume centres were defined as those that first assessed 20 or more sarcoma cases per year over the given time period of the study, whereas low-volume centres were those that saw fewer than 20. This cutoff was chosen to align with previously published data in this area [17]. 

Descriptive statistics were used to present baseline demographics for each cohort, patients treated at a high-volume centre versus a low-volume centre, respectively, and Fisher’s exact and chi-squared tests were used to evaluate for associations. Univariate and multivariate logistic regressions were used to assess for significant predictors of patient outcomes by treatment centre. The Kaplan–Meier method was used to assess progression-free and overall survival. Overall survival (OS) was defined as time from diagnosis to death or last follow-up. Disease progression was defined as time from date of diagnosis to date of earliest evidence of progression as determined by any of the following: biopsy, imaging, or clinical conclusion in the chart. Likewise, disease recurrence was defined as time from date of diagnosis to date of earliest evidence of recurrence as determined by any of the following: biopsy, imaging, or clinical conclusion in the chart. Multivariate Cox proportional hazard regression was used to assess for predictors of better survival outcomes by treatment centre as well as OS. A two-sided *p*-value < 0.05 was considered statistically significant for the multivariable analysis. All analyses were performed with StataMP, version 17.0 (StataCorp LLC, College Station, TX, USA).

## 3. Results

**Figure 1:**
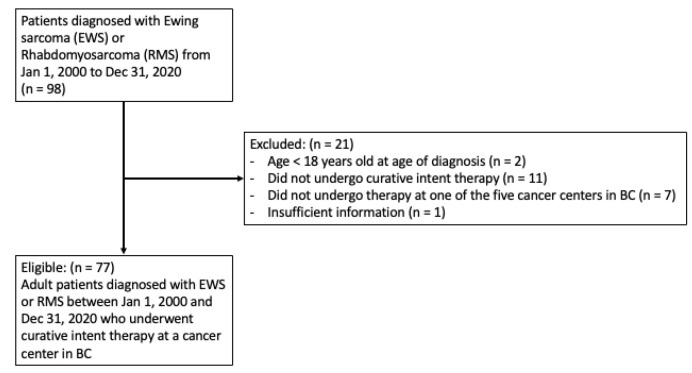
Flow diagram of the study outlining the inclusion and exclusion criteria.

Between 1 January 2000 and 31 December 2020, 98 patients with Ewing sarcoma or rhabdomyosarcoma were identified as receiving treatment in the province of British Columbia. Of the 98 patients identified, 77 patients met the inclusion criteria for the study. Forty-six of these patients were initially seen at a high-volume centre, and 31 at a low-volume centre. Figure 1 shows a flow diagram of the study illustrating the inclusion and exclusion criteria. The median length of follow-up was 3.1 years. The median date of diagnosis was December 2012 for high-volume-centre patients and September 2011 for low-volume-centre patients. 

Patients treated at high-volume centres were significantly younger than those at low-volume centres. The mean age of diagnosis was 32.1 years old at high-volume centres and 40.8 years old at low-volume centres, respectively (*p* = 0.020). There was no significant difference between other baseline patient and disease characteristics in this cohort. This includes gender, histology (Ewing sarcoma versus rhabdomyosarcoma), Charlson Comorbidity Index, ECOG performance status, or presence of metastatic disease at diagnosis. A summary of baseline patient demographics and disease characteristics is displayed in Table 1. 

In the high-volume-centre cohort, 24 (52%) patients were female and 22 (48%) were male, compared with 19 (61%) female and 12 (39%) male patients in the low-volume-centre cohort (*p* = 0.43). Thirty-four (74%) high-volume-centre patients were diagnosed with Ewing sarcoma and 12 (26%) with rhabdomyosarcoma versus 22 (71%) and 9 (29%), respectively, in low-volume centres (*p* = 0.78). Majority of the patients in both cohorts were not very comorbid, with low Charlson Comorbidity Indexes and ECOG performance status. Forty (87%) patients at high-volume centres and 22 (71%) patients at low-volume centres had a Charlson Comorbidity Index of 0. Similarly, 38 (83%) patients at high-volume centres and 21 (68%) patients at low-volume centres had an ECOG performance status of 0 or 1. Twelve (26%) of the patients at high-volume centres and 8 (26%) of patients at low-volume centres had metastatic disease at presentation (*p* = 0.98).

Significantly more patients received curative intent radiation at high-volume centres, with 29 (88%) patients receiving curative intent radiation compared with 18 (67%) at low-volume centres (*p* = 0.047). Patients seen at high-volume centres started treatment 24 days earlier on average compared with those at low-volume centres, with an average time from diagnosis to chemotherapy of 25.6 days vs. 49.8 days, respectively (*p* = 0.001). There was no significant difference regarding presentation at a multidisciplinary conference, with 37 (80%) of the patients presented at a multidisciplinary conference at high-volume centres and 28 (90%) at low-volume centres (*p* = 0.24). There was no significant difference in type of chemotherapy regimen administered at high-volume centres compared with low-volume centres (*p* = 1.00). There was no significant difference in rates of surgical intervention in high-volume centres vs. low-volume centres, 27 (59%) vs. 16 (52%), respectively (*p* = 0.54). A summary of treatment characteristics by high-volume centre vs. low-volume centre is illustrated in Table 2.

**Figure 2:**
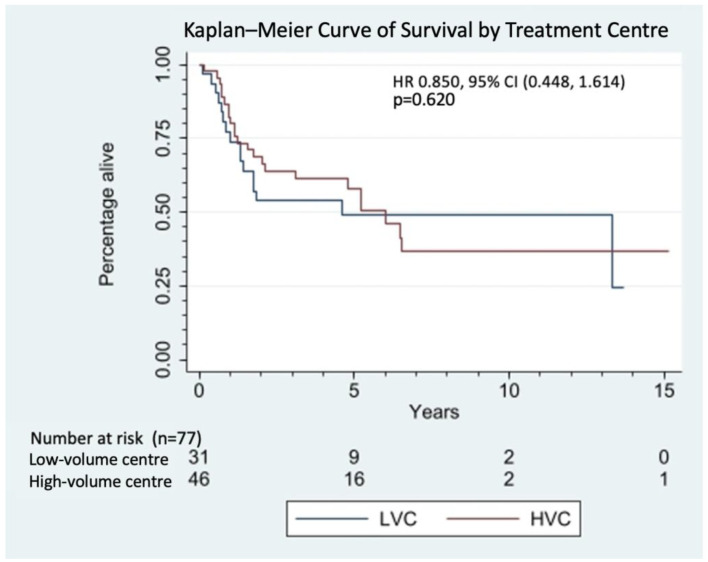
Kaplan–Meier curve of survival by treatment centre (*p* = 0.620).

There was no significant difference between disease recurrence (*p* = 0.87), disease progression (*p* = 0.49), and overall survival (*p* = 0.89) at high-volume centres vs. low-volume centres. A summary of patient outcomes by high-volume centre vs. low-volume centre is described in Table 3. Figure 2 depicts the Kaplan–Meier curve of survival by treatment-centre volume. There is no statistically significant difference in survival by treatment centre on multivariate Cox proportional hazard regression, while controlling for typical prognostic factors, such as age, ECOG performance status, disease stage at presentation, and Charlson Comorbidity Index. Undergoing a multidisciplinary sarcoma conference was not a statistically significant prognostic factor for patient survival in our cohort.

## 4. Discussion

Clinical practice guidelines recommend that management of patients with sarcoma be carried out at reference centres under specialized multidisciplinary teams due to literature demonstrating improved patient outcomes, including recurrence-free and overall survival [5,12]. These results have been validated in various European countries, including the Netherlands, France, Spain, and England, where frequent travel to and from reference centres might be more feasible than in larger geographic regions, such as British Columbia, Canada [6,7,8,9,10,11,12,13]. Our study assessed the impact of the location of diagnosis and treatment of Ewing sarcoma and rhabdomyosarcoma on patient outcomes in British Columbia. While our study demonstrated variations in care between high-volume centres and low-volume centres, it did not show any statistically significant difference in survival by treatment centre.

Patients treated at high-volume centres were on average 8 years younger than patients treated at low-volume centres. This trend could be due to several factors. First, young adults may be more willing to cope with the travel burden associated with traveling to a high-volume centre for treatment. Previous studies have shown that younger age is associated with longer travel distance to medical care, hypothesized to be due to a variety of factors, including ability to travel independently with less physical burden, fewer social and obligational ties to home community, and younger patients being more critical of the location of treatment and opting more often than older patients for a second opinion [18,19]. In addition, previous literature has demonstrated the increasing centralization of the young adult population since the early 1980s, a term coined “youthification” [20,21]. Younger adults have a higher tendency to live in central neighborhoods, as supported by the recent 2021 British Columbia census data, which found higher percentages of adolescent and young adults aged 15–39 living in large metropolitan areas, such as Vancouver and the Fraser Valley, compared with elsewhere in the province [22]. The increasing density of the adolescent and young adult population could in turn lead to a higher number of referrals to high-volume centres, which are situated in these central metropolitan areas. Lastly, adolescents and young adults with cancer are a unique patient population with specialized needs, in both cancer care and psychological, social, and economic support, for which healthcare practitioners may be more inclined to refer to a specialized centre for management [23]. All of the above reasons may contribute to the age difference seen between patients at high-volume centres vs. low-volume centres, and this lends support to increasing the availability of tailored care for adolescent and young adult patients with cancer in British Columbia, Canada.

In addition to the differences in baseline age, we found that more patients received curative radiation in high-volume centres compared with patients at low-volume centres. This is despite there being a high frequency of discussion of cases at a multidisciplinary case conference in both high- and low-volume centres, with 80% of patients at high-volume centres presented at a multidisciplinary conference and 90% at low-volume centres. Because these multidisciplinary case conferences are provincial-wide with sarcoma experts participating from across the province, the expectation would be that medical advice would be consistent regardless of home location for the patient. Studies have shown that travel burden can influence the choice of treatment for a variety of malignancies [24,25]. For example, patients who lived farther away from radiation therapy services were less likely to receive radiation for breast cancer and rectal cancer [26,27]. These findings may explain the lower rate of curative radiation seen in low-volume centres, which encompasses a geographically larger catchment area, including many rural and remote areas compared with high-volume centres. Patients in these rural or remote areas may choose not to travel from their home community to radiation facilities due to a variety of reasons, including transportation barriers, physical burden, and financial strain. This may be particularly the case regarding curative intent radiation, which is often delivered at higher doses and over a greater number of days via multiple fractions compared with palliative radiation, necessitating increased travel commitments and time away from home. In comparison, chemotherapy is more evenly distributed across the province, given that a greater number of centres have the ability to administer systemic therapy as compared with radiation therapy. In British Columbia, radiation therapy is only administered to patients with sarcoma at 5 regional cancer centres, in contrast to the over 50 clinics and centres across the province that have the capability to administer chemotherapy. These clinic/centre locations are connected via the Community Oncology Network, a collaborative partnership between BC Cancer, local hospitals, and health authorities, to ensure equitable access to cancer care throughout the province. We acknowledge that curative intent radiation is not universally recommended for patients with Ewing sarcoma and rhabdomyosarcoma, and these variations may be due to the heterogeneity of primary tumour in this cohort rather than a true differentiation. Further work is ongoing to determine if these differences are due to a deviation from multidisciplinary tumour conference recommendation versus higher use of radiation due the standard-of-care recommendation for the patient’s primary tumour site.

The average time from diagnosis to initiation of chemotherapy was significantly shorter at high-volume centres, with patients starting treatment 24 days earlier compared with their low-volume-centre counterparts (25.6 days vs. 49.8 days, respectively, *p* = 0.001). It has been shown that increased time to treatment initiation is associated with poorer survival in other cancer sites, such as breast cancer, head and neck cancer, gynecological cancer, and lung cancer [28,29]. However, there has been no consensus on the impact of time to treatment initiation in patients with sarcoma, and its relation to clinical outcomes, such as survival and morbidity, remains ambiguous [30]. Current recommendations suggest initiating treatment within 30 days of diagnosis to achieve the highest chance of cure [28]. Previous reports in the literature regarding time to treatment initiation for patients with sarcoma at various healthcare facilities around the world have ranged from an average of 21 to 43 days, depending on the healthcare region, which is comparable to the values we found in our study [28,31,32,33]. There were several factors that often contributed to time to treatment initiation in our cohort. One common cause was diagnostic uncertainty. Due to their rarity and heterogeneity, accurate diagnosis of soft tissue sarcoma can be difficult for nonspecialized pathologists [34,35]. All patients with sarcoma in British Columbia undergo expert pathology review at a high-volume centre, and molecular confirmation is nearly universally required for Ewing sarcoma prior to treatment. Sarcoma pathologists in British Columbia establish the molecular diagnosis of Ewing sarcoma and fusion-positive rhabdomyosarcoma with the nano-string-based assay with a turnaround time of up to 1 week. Oftentimes there can be delay in the transportation of tumour samples from more distant outside centres, leading to a delay in establishing the diagnosis and initiating treatment [36]. Other factors contributing to time to treatment initiation included waiting for pretreatment investigations, such as staging scans, awaiting specialist referral, and patient preference. The increased interval between diagnosis and treatment at low-volume centres compared with high-volume centres could reflect inequality in access to health resources. Disparities in resource utilization may lead to longer wait times for the necessary investigations and appointments prior to treatment. Additionally, correspondence with a sarcoma specialist stationed elsewhere in the province, such as Vancouver, to aid in the coordination of complex chemotherapy regimens may also add to wait times. This is consistent with prior literature, which cites transitions in care between institutions as responsible for the greatest increases in time to treatment initiation [33]. These data can act as an incentive for future quality improvement projects to further improve access to care.

Despite the differences in patient characteristics and treatment variation seen in high-volume centres vs. low-volume centres, there was no statistically significant difference in overall survival by treatment centre. This is in contrast to prior literature from mostly European countries, which has shown improved patient outcomes when managed at a centre of expertise. Although several studies conducted in the United States (US), a country more geographically similar in size to Canada, have shown better patient outcomes if managed at centres of expertise, other conflicting US-based studies have also demonstrated that patients treated at reference centres, such as National Cancer Institute–Designated Cancer Centres (NIC-CCs), have similar morbidity and long-term survival compared with other non–National Cancer Institute–designated hospitals [13,37,38,39]. The lack of significant difference in patient outcomes seen in our study may be explained by British Columbia’s existing hub-and-spoke model of care. A unique aspect of cancer care in British Columbia is that it is provincially provided through a comprehensive cancer program. All radiation therapy services in British Columbia are provided through BC Cancer, as well as the majority of chemotherapy. Each of the BC Cancer centres delivers cancer therapy based on provincial guidelines and standards established by BC Cancer. This is in contrast to other provinces, such as Ontario, where cancer services are delivered through 14 Regional Cancer Programs, each of which may have its own practice patterns [40]. As well, this healthcare model allows multidisciplinary tumour boards to be run provincially with experts across the province affiliated with BC Cancer participating, which may not be the case for regional cancer programs where tumour boards may be limited to the local expertise in the program. British Columbia’s hub-and-spoke model of care is an organizational design that arranges healthcare delivery into a network of services consisting of anchoring centres (hubs) offering the full spectrum of care, complemented by secondary centres (spokes) with more limited services. This enables cancer care in British Columbia to be provincially determined and regionally delivered, allowing for more consistencies in healthcare delivery compared with other models. With this model, low-volume-centre colleagues can easily seek help and support from their high-volume-centre counterparts, allowing for an increased ease of communication, collaboration, and multidisciplinary care. We hypothesize that increased consistencies in cancer care delivery across the province afforded by British Columbia’s hub-and-spoke model of care may explain the lack of significant difference in patient outcomes seen in our study, in contrast to other healthcare regions, which may not necessarily have this model in place. Perhaps these findings can aid other geographically large centres when organizing healthcare delivery networks.

We acknowledge that the small patient cohort size poses limitations to our study. The small sample size is due to the rarity of Ewing sarcoma and rhabdomyosarcoma and the need to evaluate a homogenously-cared-for province of patients. The small cohort size lends itself to higher variability and potential type II error, which can make it difficult to interpret results and obtain statistical significance. It is possible that the lack of statistical significance in patient outcomes by treatment centre observed in our study may be due to the small sample size. We have tried to mitigate this by considering a longer time frame of patients; however, the long time frame considered also may have introduced its own potential mixed factors to our data, such as changes in therapeutic techniques, efficiency of public communications, and life expectancy. As our study is a retrospective cohort study, it may be susceptible to confounders and experience inaccurate or incomplete chart documentation, and it is unable to determine causation, only association. The possible confounders of this cohort study include factors such as socioeconomic status (SES); however, given the limited sample size and retrospective nature of the study, SES was difficult to capture. Lastly, the classification of a reference centre or high-volume centre is currently not well defined in the literature and individual to each healthcare system. Similar to our study, most prior studies used various volume thresholds to distinguish between high-volume centres vs. low-volume centres [6,17,41,42]. Others have used alternate indicators, such as affiliation with universities or academic sites, or statistical analysis to identify a meaningful threshold [9,43]. However, there has been no consensus in clinical practice guidelines or in the literature regarding the definition of a high-volume centre, leaving an important area of future consideration.

In summary, variations in treatment characteristics between patients with Ewing sarcoma and rhabdomyosarcoma treated at high-volume centres vs. low-volume centres may reflect regional differences in access to care and practice patterns; however, we did not identify a difference in patient outcomes by treatment centre. This may be explained by British Columbia’s existing hub-and-spoke model of care, which allows for greater consistencies in healthcare delivery across the province compared with other models. Results of this study can be used to better inform decisions regarding triaging and centralization of patients with curable Ewing sarcoma and rhabdomyosarcoma in British Columbia, Canada, and may help in healthcare resource planning for other regions spanning a wide geographical area.

## Figures and Tables

**Table 1:** Baseline patient demographics and disease characteristics by high-volume centre (HVC) vs. low-volume centre (LVC).

		LVC (*n* = 31)	HVC (*n* = 46)	*p*-Value
Age		40.8	32.1	0.020
Gender	Female	19 (61%)	24 (52%)	0.43
	Male	12 (39%)	22 (48%)	
Histology	EWS	22 (71%)	34 (74%)	0.78
	RMS	9 (29%)	12 (26%)	
Charlson comorbidity Index	Zero	22 (71%)	40 (87%)	0.082
	One or Higher	9 (29%)	6 (13%)	
ECOG status	0	6	21	0.15
	1	15	17	
	2	4	4	
	3	4	3	
Metastatic disease at presentation	No	23 (74%)	34 (74%)	0.98
	Yes	8 (26%)	12 (26%)	

**Table 2:** Treatment characteristics by high-volume centre (HVC) vs. low-volume centre (LVC).

		LVC (*n* = 31)	HVC (*n* = 46)	*p*-Value
Multidisciplinary tumour Board discussion	No	3 (10%)	9 (20%)	0.24
	Yes	28 (90%)	37 (80%)	
Chemotherapy	VDC/IE Q2W *	6 (19%)	9 (20%)	1.00
	VDC/IE 3W **	21 (68%)	31 (67%)	
	Other	4 (13%)	6 (13%)	
Curative radiation	No	9 (33%)	4 (12%)	0.047
	Yes	18 (67%)	29 (88%)	
Surgery	No	15 (48%)	19 (41%)	0.54
	Yes	16 (52%)	27 (59%)	
Mean days diagnosis to chemotherapy		49.8	25.6	0.001

* VDC/IE Q2W—Etoposide, Ifosfamide–Mesna alternating with vinCRIStine, DOXOrubicin, and Cyclophosphamide at TWO weekly intervals. ** VDC/IE Q3W—Etoposide, Ifosfamide–Mesna alternating with vinCRIStine, DOXOrubicin, and Cyclophosphamide at THREE weekly intervals.

**Table 3:** Patient outcomes by high-volume centre (HVC) vs. low-volume centre (LVC).

		LVC (*n* = 31)	HVC (*n* = 46)	*p*-Value
Disease recurrence	No	21 (68%)	32 (70%)	0.87
	Yes	10 (32%)	14 (30%)	
Disease progression	No	25 (81%)	34 (74%)	0.49
	Yes	6 (19%)	12 (26%)	
Survival	Alive	15 (48%)	23 (50%)	0.89
	Diseased	16 (52%)	23 (50%)	

## Data Availability

The data are not publicly available due to patient privacy regulation and ethical restriction.
